# Genome-Wide Identification, Characterization, and Stress-Responsive Expression Profiling of Genes Encoding LEA (Late Embryogenesis Abundant) Proteins in Moso Bamboo (*Phyllostachys edulis*)

**DOI:** 10.1371/journal.pone.0165953

**Published:** 2016-11-09

**Authors:** Zhuo Huang, Xiao-Juan Zhong, Jiao He, Si-Han Jin, Han-Du Guo, Xiao-Fang Yu, Yu-Jue Zhou, Xi Li, Ming-Dong Ma, Qi-Bing Chen, Hai Long

**Affiliations:** 1 College of Landscape Architecture, Sichuan Agricultural University, 211 Huimin Road, Wenjiang, Chengdu 611130, Sichuan, China; 2 Chengdu Institute of Biology, Chinese Academy of Sciences, Chengdu 610041, Sichuan, China; NARO Hokkaido Agricultural Research Center, JAPAN

## Abstract

Late embryogenesis abundant (LEA) proteins have been identified in a wide range of organisms and are believed to play a role in the adaptation of plants to stress conditions. In this study, we performed genome-wide identification of LEA proteins and their coding genes in Moso bamboo (*Phyllostachys edulis*) of Poaceae. A total of 23 genes encoding LEA proteins (PeLEAs) were found in *P*. *edulis* that could be classified to six groups based on Pfam protein family and homologous analysis. Further *in silico* analyses of the structures, gene amount, and biochemical characteristics were conducted and compared with those of *O*. *sativa* (OsLEAs), *B*. *distachyon* (BdLEAs), *Z*. *mays* (ZmLEAs), *S*. *bicolor* (SbLEAs), *Arabidopsis*, and *Populus trichocarpa*. The less number of PeLEAs was found. Evolutionary analysis revealed orthologous relationship and colinearity between *P*. *edulis*, *O*. *sativa*, *B*. *distachyon*, *Z*. *mays*, and *S*. *bicolor*. Analyses of the non-synonymous (Ka) and synonymous (Ks)substitution rates and their ratios indicated that the duplication of *PeLEAs* may have occurred around 18.8 million years ago (MYA), and divergence time of LEA family among the *P*. *edulis*-*O*. *sativa* and *P*. *edulis*–*B*. *distachyon*, *P*. *edulis-S*. *bicolor*, and *P*. *edulis-Z*. *mays* was approximately 30 MYA, 36 MYA, 48 MYA, and 53 MYA, respectively. Almost all *PeLEAs* contain ABA- and (or) stress-responsive regulatory elements. Further RNA-seq analysis revealed approximately 78% of *PeLEAs* could be up-regulated by dehydration and cold stresses. The present study makes insights into the LEA family in *P*. *edulis* and provides inventory of stress-responsive genes for further functional validation and transgenic research aiming to plant genetic improvement of abiotic stress tolerance.

## Introduction

As sessile organisms, plants have evolved a wide spectrum of adaptations to cope with the inevitable challenges of environmental stress, such as drought, high salinity, and cold, etc. Many aspects of these adaptation processes, including developmental, physiological and biochemical changes, are regulated or achieved by stress-responsive gene expression. The late embryogenesis abundant (LEA) proteins constitute of a family of hydrophilic proteins that are presumed to play a protective role during exposure to different abiotic stresses. They were first described to highly accumulate during the late stages of cotton seed development, when the embryo becomes desiccation tolerant [1]. They were not only found in the seeds of many other plants, but also detected in vegetative organs. More importantly, they are usually induced under stress conditions such as cold, drought, or high salinity [2, 3].

LEA proteins were initially classified to six subgroups on the basis of specific domains [4]. With increasing information on family members, expression profile differences, derived organisms and also the development of bioinformatic tools, the classification has been subjected to different rearrangements [5–10]. Many studies have been performed to characterize their functions, especially the roles in stress responses. LEA25, a group 4 LEA protein from tomato (*Solanum lycopersicum*), can improve tolerance against high salinity and freezing when expressed in *Saccharomyces cerevisiae* [11]; Overexpression of barley (*Hordeum vulgare*) *HVA1* in wheat (*Triticum aestivum*) and rice (*Oryza sativa*) confers enhanced drought tolerance [12, 13]. Virus-induced silence of *HVA1* and *DHN6* resulted in significant decrease in drought tolerance [14]. JcLEA, a abscisic acid (ABA) and stress-induced group 5 LEA protein of *Jatropha curcas*, could enhance tolerance to drought and salt stress in *Arabidopsis* [15]. Overexpression of a maize group 3 LEA gene, *ZmLEA3*, in tobacco and yeast conferred tolerance to osmotic and oxidative stresses [16]. Results from these studies suggest that *LEA* family could be considered as a reservoir for stress-responsive genes, which have great potential in genetic improvement of stress tolerance in plants.

The rapid generation of plant whole genome sequences provides opportunities to genome-widely identify and classify the genes encoding LEA proteins in plant, which will not only provide insights into evolution of LEA family, but also provide basis for further systematically expression profiling, and in-depth biochemical, functional and physiological studies. To date, the genome-wide characterization of LEA family has been performed in several genome-sequenced plant species, such as *Arabidopsis* [6, 8], *Populus trichocarpa* [17], legumes [10], *O*. *sativa* [18], and *Brachypodium distachyon* [19].

Bambusoideae, generally called bamboo, belongs to grass family (Poaceae) and is comprised of more than 1,400 species. Unlike other herbaceous species of Poaceae, the major components of Bambusoideae are arborescent and perennial woody species, which live exclusively in forests and grow large woody culms up to 30 cm in diameter and 12 m in height [20]. Fast growing, high productivity, strong regeneration capability make it one of the most important non-timber forest resources in the world. According to the statistics, about 2.5 billion people depend economically on bamboo, and the annual international trade in bamboo amounts to over 2.5 billion US dollars [21]. Despite of its economic importance, little is known about its responses to abiotic stress and underlying mechanism at molecular level. This might be partly due to the lack of genomic resources. Recently, the genome of Moso bamboo (*Phyllostachys edulis*), a large woody bamboo with high ecological and economic values, were decoded [22]. In this study, we searched the *P*. *edulis* genome to identify the genes encoding LEA proteins (*PeLEAs*). *In silico* analyses of promoter elements, biochemical properties, and evolutionary features were performed. Further RNA-seq based expression profiling was also conducted to investigated their responses to dehydration and cold.

## Materials and Methods

### Data resources

The whole genome dataset, full length cDNA, and EST of *P*. *edulis* are downloaded from the Bamboo Genome Database (www.bamboogdb.org) and National Center for Biotechnology Information (www.ncbi.nlm.nih.gov/). They consist of 31,987 protein-coding genes predicted from whole genome sequences, and 10,608 low redundant full-length cDNA (FLcDNA) sequences and 38,000 ESTs from leaf, shoot, and seedling libraries. The LEA genes in *A*. *thaliana* genome were according to Hundertmark and Hincha [8], and their sequences were obtained from The Arabidopsis Information Resource (www.arabidopsis.org). The *O*. *sativa* and *B*. *distachyon* genome data were also obtained from the website of Rice Genome Annotation Project (*O*. *sativa*.plantbiology.msu.edu), and the genome data of *Zea mays* and *Sorghum bicolor* was obtained from EnsemblPlants (plants.ensembl.org/)

### Identification of genes encoding LEA protein from P. edulis genome

All LEA protein sequences of *A*. *thaliana* [8] were used as queries to Blast search against whole genome dataset of *P*. *edulis* with expectation value of 0.01. The resulted sequences were analyzed by Pfam database [23] to characterize obtained sequences into Pfam family. The full length cDNA and EST datasets were queried for further evidences for the obtained genes.

### *In silico* analyses of of LEAs

The grand average of hydropathicity index (GRAVY), theoretical isoelectric point (pI) and molecular weight were analyzed by using the ProtParam Tool (web.expasy.org/protparam/).

The coordinates of exon and intron of LEA genes of *P*. *edulis* were extracted from their corresponding scaffolds and exon-intron structures were illustrated using Gene Structure Display Server (GSDS, http://gsds.cbi.pku.edu.cn/) [24].

Sequences of 1500 nt upstream of the coding sequences were retrieved from bamboo genome database (http://www.bamboogdb.org/). The putative *cis*-acting elements related to abiotic stress response were analyzed by querying the PLACE database ((http://www.dna.affrc.go.jp/PLACE/) [25].

### Evolutionary analyses of the paralogues and orthologues in four grass species

Reciprocal BLASTP was carried out to establish orthologous relationship among *P*. *edulis*, *O*. *sativa* and *B*. *distachyon*. The hits threshold values were set as E-value <1e-10, score >200, and positive >70%. The paralogous relationship within *P*. *edulis* was also analyzed with more stringent parameters of E-value <1e-50, score >200, and positive >80%. The synonymous (Ks) and non-synonymous (Ka) substitution rates of paralogues and orthologues were analyzed by Ka_Ks calculator 2.0 [26]. Time (million years ago, MYA) of duplication and divergence was calculated using a synonymous mutation rate of one substitutions per synonymous site per year as T = Ks/2λ (λ = 6.5×10^−9^) [27, 28].

### Stress treatment and expression profiling

To evaluate expression patterns of *PeLEA* under abiotic stress, dehydration and cold treatments were conducted. The full young unexpanded leaves were detached from different *P*. *edulis* plants with similar growth status. For dehydration treatment, the whole leaves were placed on the dry filter paper and treated under room temperature (20°C and 50% humidity). For cold treatment, the leaves were put into a chamber set to 0°C without light. At 2h and 8h after each treatment, ten individual leaves were immediately frozen in liquid nitrogen and the total RNA of was extracted according to the manual of the TRIZOL RNA Kit (TIANGEN, Beijing, China). The same amount of untreated leaves were also sampled and used as control. The qualities and quantities of extracted nucleotide were measured by NanoDrop 2000 Spectrophotometer (Thermo Fisher, USA) and Agilent 2100 RNA 6000 Nano kit. The threshold of the quality of extracted RNA was RIN ≥ 7 with concentration ≥ 150 ng/ul and amount ≥ 5 ug.

The cDNA library construction and sequencings on Illumina HiSeq^™^ 4000 platform were performed by Onmath Co.(Chengdu, China), following the manufacturer’s standard protocol. The 150 bp sequences by pair-end sequencing were generated as raw data. The filtered clean reads were mapped to all obtained *PeLEA* by using TopHat v2.0.9. HTSeq v0.6.1 was used to count the reads numbers mapped to each gene. And then RPKM of each gene was calculated based on the length of the gene and reads count mapped to this gene. RPKM, Reads Per Kilobase of exon model per Million mapped reads, considers the effect of sequencing depth and gene length for the reads count at the same time, and is currently the most commonly used method for estimating gene expression levels [29].

## Results and Discussions

### Identification and classification of LEA genes in *P*. *edulis*

By blast query of gene model of *P*. *edulis* genome, 23 putative LEA proteins were found. By Pfam family domain analysis, 21 candidates could be assigned to LEA family, in which 6 were supported by known FLcDNA or EST (S1 Table). Six groups of LEA protein were identified by homology to known LEA proteins. We here classified them based on the Pfam nomenclature, as it is specifically related to conserved domains [8] and integrate sequences from diverse species. The correspondence of different nomenclatures or classifications of LEA family was present in S2 Table. Only one gene was found in groups LEA_1 and LEA_6 (also known as PvLEA18); Four LEA2 proteins were detected; Both group LEA_3 and group LEA_4 were comprised of five proteins, and six proteins are assigned as dehydrins. However, no gene was identified as groups LEA_5, SMP, or AtM (S1 Table) in *P*. *edulis*.

**Fig 1:**
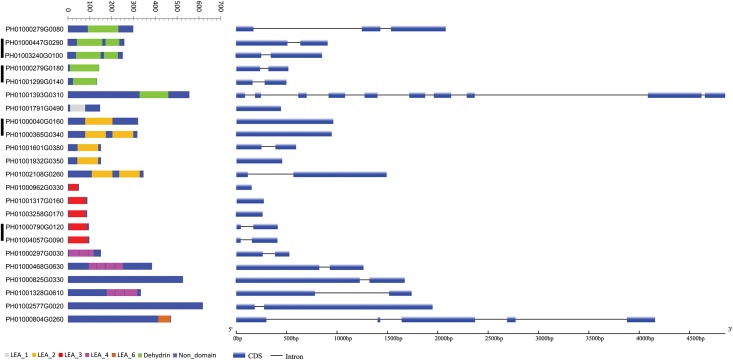
Protein domain organization (left column) and exon-intron structure (right column) of *PeLEAs*. The present domains were identified by Pfam database. Different color boxes indicate the conserved domain or non-domain region on LEA proteins in of LEA groups. Black bars indicate paralogous gene pairs.

Members of groups LEA_1, LEA_3, and LEA_6, and four members of dehydrin, and three LEA_2 members have a single LEA domain; two members of dehydrin and LEA_2 contain two repetitive domains, and three members of LEA_4 contain three or four domains (Fig 1a). No conserved domains were found in two candidates PH01000825G0330 and PH01002577G0020. However, they showed high degree of homology to LEA_4 proteins from Arabidopsis, AT1G72100.1 (E-value = 6e-062) and AT2G42560.1 (E-value = 1e-025), respectively. Therefore, we also assigned the two PeLEA proteins into group LEA_4. Seven *LEAs* of *P*. *edulis* are comprised of a single uninterrupted coding region, whereas 16 members are composed of two to ten exons and one to nine introns (Fig 1b).

By BLAST search, we found five *PeLEAs* are homologous to at least 13 functionally known LEA genes associated with abiotic stress tolerance (S3 Table), indicating that they might be considered as candidate genes for drought and cold tolerance.

### Comparison of gene amount and protein characteristics

*P*. *edulis* belongs to grass family (Poaceae). It is interesting to compare the LEA families among the sequenced species of Poaceae. Filiz et al. identified 36 LEA genes in *B*. *distachyon* [19]. However, these genes were obtained by BLAST search using only a representative of *LEA2*-*LEA6* of *Arabidopsis* as queries. This might lose sight on some LEAs, such as seed mature protein (SMP). Additionally, the LEA gene number of *O*. *sativa* varies in different studies [17, 18]. Therefore, we re-searched LEAs in the two species by the same strategy used in this study. As a result, 46 LEA genes were identified in *B*. *distachyon* (S1 Table), and 35 LEA genes were found in *O*. *sativa* (S1 Table), respectively. Additionally, we also search the high quality genome data of *Z*. *mays* and *S*. *bicolor*, by which 35 and 30 ZmLEAs and SbLEAs were found. They were also classified by Pfam and homology analyses according to Pfam family nomenclature (S1 Table).

**Fig 2:**
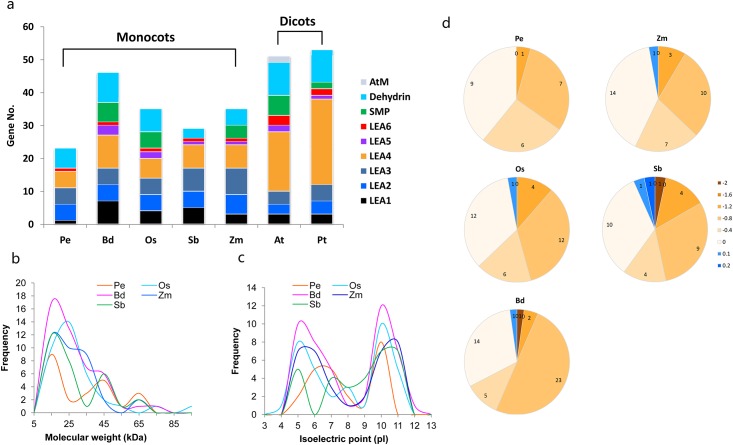
Comparisons of gene amount (a), molecular weight (b), isoelectric point (c), and grand average of hydropathicity index (GRAVY, d).

We then compared the gene amount of different LEA groups between *P*. *edulis*, and four Poaceae species, as well as two well studied dicot representatives *A*. *thaliana* [6, 8] and *P*. *trichocarpa* [17]. Comparing to five monocot species, two dicots have more LEA proteins in their genome. *P*. *edulis* contains the least number of LEAs among the seven species; *O*. *sativa* and *Z*. *mays* share similar LEAs number, and *B*. *distachyon* contains the most abundant LEA proteins in five monocots analyzed (Fig 2a). Dehydrin, LEA_1, LEA_2, LEA_3, LEA_4, and LEA_6 are common groups in all species. Dicots rich in LEA_4, accounting for more than 35% and 49% of LEA family members in *A*. *thaliana* and *P*. *trichocarpa*, respectively. As mentioned above, no LEA_5, SMP, or AtM were found in *P*. *edulis*. The AtM are only found in *A*. *thaliana*.

Most of the *PeLEA* genes encode rather small proteins, in which the deduced molecular weights (MW) of ~61% members are less than 35 kDa. Similarly, ~70%- ~88% of LEAs in other four monocots are smaller than 35 kDa (Fig 2b). The theoretic pI values of PeLEAs range from 4.81 to 9.86, and the other four monocots show similar pI ranges from ~4.0 to 11. Approximately ~50% of the LEA proteins of *P*. *edulis*, *O*. *sativa*, *B*. *distachyon*, and *Z*. *mays* are more than 7.0. Whereas 70% of SbLEAs have pI more than 7.0, which is significantly higher than those in other four monocot species (Fig 2c).

We also calculated the grand average hydropathicity (GRAVY) index of LEAs. All PeLEAs show negative values, indicating that PeLEAs are all hydrophobic. Among the OsLEAs, BdLEAs, ZmLEAs, and SbLEAs, only one or two LEA proteins show GRAVY values larger than 0 (Fig 2d, S1 Table). This is similar as those reported in dicots previously [8,17], suggesting the apparently hydrophobic characteristic of LEA proteins in plants.

### Orthologous and paralogous relationship among Poaceae

**Fig 3:**
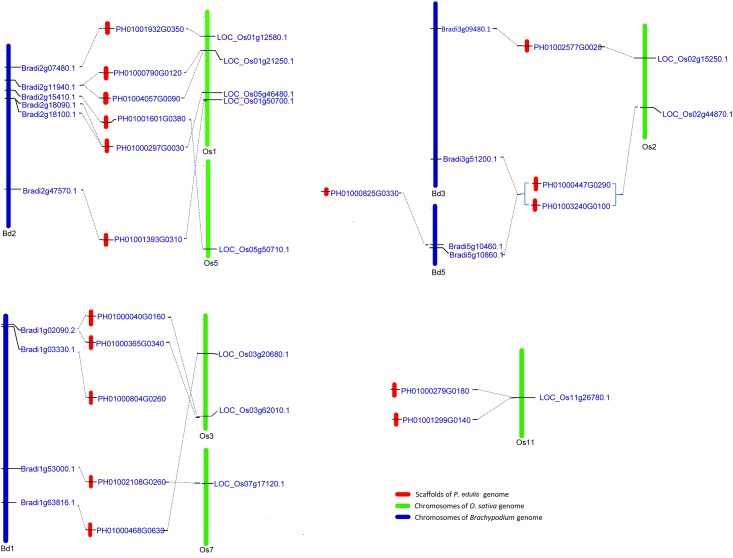
Genome organization, orthologous relationship and colinearity of *LEAs* among *P*. *edulis* (Pe), *O*. *sativa* (Os), and *B*. *distachyon* (Bd). Only the orthologues are present. The blue and green bars indicate chromosomes of *B*. *distachyon* and *O*. *sativa*, respectively. Their corresponding chromosomes numbers were showed at the bottom of chromosomes. The scaffolds on which *PeLEAs* are located are represented by red bars.

We conducted reciprocal BLASTP analysis of the orthologous relationship between five monocot species. A total of 11 *OsLEAs* (~31.4%), 14 *BdLEAs* (~30.4%), 14 *ZmLEAs* (~40%), and 11 *SbLEAs* (~36.7%) were found to be orthologous to 18 *PeLEAs* (~78.3%) (S4 Table), respectively. Most of the orthologues of *PeLEAs* and *OsLEAs* were located in the *P*. *edulis*-*O*. *sativa* colinearity regions [22]. The orthologous *OsLEAs* and *BdLEAs* were mainly distributed on six and four chromosomes, respectively, and also shared same syntenic patterns as revealed previously [30] (Fig 3).

Genome duplications, such as tandem and segmental duplications usually give rise to gene copy numbers. The reciprocal BLASTP analysis identified four paralogous pairs of *PeLEAs* (S4 Table). Previous study proposed that the monocot chromosomes, such as *O*. *sativa* (12 pairs of chromosomes) and *B*. *distachyon* (five pairs chromosomes), were derived from an intermediate with 12 pairs of chromosomes [30]. *P*. *edulis* has 24 pairs of chromosomes and sequencing of its genome revealed that it carries as two duplicates as that of rice gene model sets [22]. However, only four duplicated *PeLEAs* to orthologous *OsLEAs* were detected. Additionally, *P*. *edulis* contains the least PeLEAs gene number among the species analyzed. These results suggested that LEA family in *P*. *edulis* may have undergone significantly gene loss during evolution.

### Divergence rates and selection

**Fig 4:**
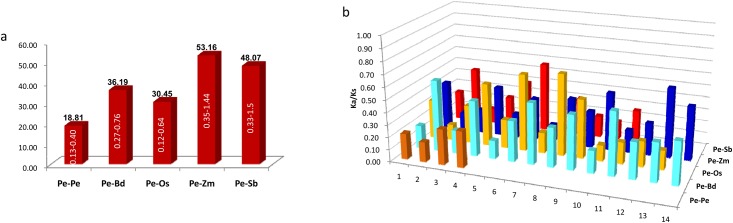
Ks (a), divergence time (a) and Ka/Ks (b) distributions of paralogous of *P*. *edulis*, and between *P*. *edulis*-*O*. *sativa* (Os), *P*. *edulis-B*. *distachyon*, *P*. *edulis-Z*. *mays*, and *P*. *edulis-S*. *bicolor* orthologue pairs.

In order to evaluate the timing of intragenomic gene duplication events, as well as divergence of orthologues, the synonymous substitution rate (Ks) was calculate. The paralogous gene pairs exhibited mean Ks of 0.24. Then estimated by universal substitution rate of 6.5 × 10^−9^ mutations per site per year, the duplications of *PeLEAs* may occur around 18.8 million years ago (MYA) (Fig 4a and 4b, S5 Table). This is different from the estimated timing of whole genome duplication at 7-12MYA [22], as well as divergence time of AP2/ERF transcription factors superfamily of around 15 MYA [31].

Among the *P*. *edulis*-*O*. *sativa*, *P*. *edulis*–*B*. *distachyon*, *P*. *edulis*-*S*. *bicolor* and *P*. *edulis*-*Z*. *mays* orthologous gene pairs, the mean Ks of are ~0.40, ~0.47, ~0.62, and ~0.69, indicating that the divergent time of LEAs families among these species was approximately 30 MYA, 36 MYA, 48 MYA, and 53MYA, respectively (S5 Table, Fig 4a). This result is also different from previous estimation of divergence time within Poaceae. By using whole-genome sequences of chloroplasts, Wu and Ge estimated the divergence time of three subfamilies, Bambusoideae (three bamboo species), Pooideae (nine species including *O*. *sativa*, *O*. *nivara*, etc.) and Ehrhartoideae (five species including *Triticum aestivum*, *H*. *vulgare*, *B*. *distachyon*, etc.) of Poaceae [32]. They showed that Ehrhartoideae diverged from the clade of Bambusoideae and Pooideae at approximately 46.98 (40.80–51.60) MYA, whereas the Bambusoideae and Pooideae clades split at ~42.80 (36.61–48.80) MYA [32]. Similar results obtained by calculating the Ks of 968 single-copy gene clusters, indicating that the mean Ks for *B*. *distachyon*–*P*. *edulis*, *O*. *sativa*–*P*. *edulis*, *Sorghum*–*P*. *edulis* and Z. mays–*P*. *edulis* are 0.61, 0.63, 0.76, and 0.84, and their divergence time is around 46.9MYA, 48.6MYA, 58.8MYA, and 64.6 MYA, respectively [22]. These differences may be due to that the LEA proteins are not highly conserved and might not be essential for surviving, and therefore they may have different substitution rates to the universal rate.

**Fig 5:**
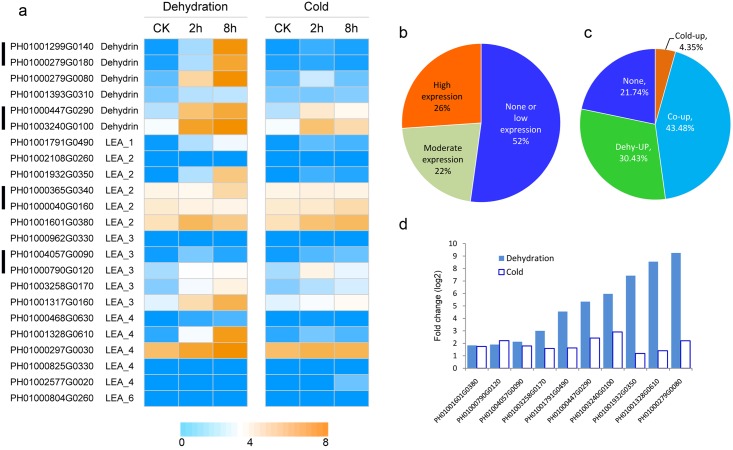
Expression profiling of *PeLEA* family under dehydration and cold stresses. a, Heatmap of expression values (showed by log_2_ RPKM values) in control, and in treated samples 2h and 8h after treatment; b, Percentage of genes in different expression levels under normal conditions; c, Percentage of genes exhibiting different responses to dehydration and cold; d, Comparison of fold changes of co-upregulated genes under dehydration and cold stresses. The expression data is obtained through two biological replicates. The relatedness of two replicates was present in S1 Fig. The black bars on the left indicate paralogous genes.

We also calculated the ratios of non-synonymous (Ka) versus synonymous (Ks) substitution rate (Ka/Ks) for duplicated gene-pairs as well as the orthologues of *O*. *sativa*, *B*. *distachyon*, *Z*. *mays*, and *S*. *bicolor* (Fig 5c). The Ka/Ks ratio is a measure of the selection pressure to which a gene pair is subjected. Ka/Ks < 1 means purifying or negative selection, Ka/Ks = 1 stands for neutral selection, and Ka/Ks > 1 indicates positive selection [27]. The Ka/Ks for paralogous gene pair of *PeLEAs* is 0.16 to 0.30 with mean of ~0.24. Those for orthologous gene pairs of *PeLEAs-OsLEAs*, *PeLEAs-BdLEAs*, *PeLEAs-SbLEAs*, and *PeLEAs*-*ZmLEAs* are 0.13 to 0.67 with mean of ~0.34, 0.15 to 0.58 with mean of ~0.34, 0.12 to 0.57 with mean of 0.28, and 0.14 to 0.57 with mean of 0.32, respectively (Fig 4b). These results indicated that they appear to have undergone extensive purifying selection during evolution.

### The *cis-acting* regulatory elements and stress-induced expression

The *cis*-acting elements in the promoter region are short motifs on which the transcription factors could bind on to regulate their expressions. The ABRE (ABA responsive element) plays a key role in ABA signaling during seed development and under abiotic stresses, while the DRE/CRT/LTRE (drought responsive/C-repeat/low temperature response) is well known to be involved in drought-, cold- and high-salt-responsive gene expression regulated by CBF/DREB1 transcription factors [33, 34]. The two motifs are predominantly present in LEA genes [18, 8]. To identify putative stress-responsive *LEAs* in *P*. *edulis*, we queried both motifs in the -1500 nt promoter region of 23 *PeLEAs* by PLACE database. Almost all the PeLEAs contain both of motifs in their promoters.

We further performed RNA-seq to evaluate dynamic expression levels of *PeLEA* genes under dehydration and cold stresses (Fig 5a). Approximately 4 giga bases high quality data (Q30>92%) for each sample were generated and used to calculate RPKM values of *PeLEAs*. It indicated that 26% of *PeLEAs* highly expressed (log2 RPKM value >4) in leaf under normal conditions, 22% showed moderate expression (log2 RPKM value between 1 to 4), and the remaining 52% are in very low expression level or not expressed (Fig 5a and 5b). In *Arabidopsis*, 22 *LEAs* showed highly expression levels in non-seed tissue under non-stressful condition, in which 10 (19.6%) highly expressed in leaf [8]. Three out of six highly-expressed *PeLEAs* belong to LEA_2 group, and one of *dehydrin*, *LEA_3* and *LEA_4* exhibited high expression levels. This results suggest that members of group LEA_2 in *P*. *edulis* might play important roles in development.

A total of 17 *PeLEAs* were upregulated (RPKM fold change>2) by dehydration, and none was downregulated. Under cold treatment, 11 genes were upregulated and only no gene was downregulated. Ten genes cold be upregulated by both dehydration and cold stresses. Only five genes kept in constant expression level or didn’t expressed (Fig 5c) under the two treatments. Interestingly, most of the co-upregulated genes are much more sensitive to the dehydration than to cold (Fig 5d), but opposite condition appears in *Arabidopsis*. These results suggest that the stress-induced responses of LEA family may be divergent between *P*. *edulis* and *Arabidopsis*.

We analyzed expression patterns of paralogous *PeLEAs*. Gene duplication is one of the resources of pseudogenes, as an intact functional copy still exists and then the function loss of a duplicated gene only has little effect on an organism's fitness. According to the transcriptome data, all four paralogous gene pairs showed detectable expression levels, indicating that they might not be pseudogenes Three out of the four paralogous gene pairs share highly conserved expression patterns during dehydration and cold stresses (Fig 5a). We also noticed that these paralogues also contain conserved intron/exon structures (Fig 1). These results indicated the functional and structural conservation during evolution.

Dehydrin is the most characterized group of LEA proteins and accumulate during seed desiccation and in response to water deficit induced by drought, low temperature or salinity [35–37]. All six *dehydrins* of *P*. *edulis* were induced by dehydration, and four of them were also induced by cold ((Fig 5a). This is quite different from those in *Arabidopsis*, that five of 10 *dehydrins* are responsive to cold and only two are induced by drought [8].

Both *P*. *edulis* and *Arabidopsis* (*At5g06760*, previously known as *LEA4-5*) has one *LEA* of LEA_1 group which is more sensitive to dehydration or dehydration. This gene has been reported to be responsive to water deficit and its overexpression leads to tolerance to severe drought in *Arabidopsis*. Four of five genes in LEA_3 group are positively responsive to dehydration, and 3 of them are also responsive to cold. *Arabidopsis* has three stress-induced LEA_3: *At1g02820* and *At4g15910* (*Drought-induced 21*, *DI21*) are responsive to both of drought and cold, *At4g02380* (*Senescence-associated gene 21*, *SAG21*) is regulated by cold, respectively. Two genes of LEA_4 (PH01000297G0030 and PH01001328G0610) are significantly upregulated by dehydration, in which later is more sensitive and is also induced by cold. In *Arabidopsis*, LEA_4 is in predominant number among the AtLEA family (18 of 51), but only two of them, *At2g42530* (*Cold-regulated 15B*, *COR15B*) and *At2g42540* (*COR15B*) are upregulated by cold [8]. *P*. *edulis* has only one LEA_6 (also known as PvLEA18) and its expression is not detectable in leaf both in normal and stressful conditions.

Three of five *LEA_2* are upregulated by dehydration and two of them are also responsive to cold. Previous studies classified this group as 'atypical' LEA proteins because of their more hydrophobic character [38, 39]. Although little is known of their function, some reports indicated that they will accumulate in response to diverse stresses in plants, such as cotton (*LEA14-A*) [38], *Craterostigma plantagineum* (*PcC27-45*) [40], soybean (*D95-4*) [41], tomato (*ER5*) [42], and *Arabidopsis* (LEA14) [43], etc. Overexpression of *CaLEA6* in tobacco improves tolerance to dehydration and NaCl [44]; Transgenic sweetpotato non-embryogenic calli that overexpressed *IbLEA14* showed increased tolerance to drought and salt stress by enhancing lignification [45]; Overexpression of *SiLEA14* foxtail millet improved tolerance to salt and drought [46]. All these results suggest that LEAs of group LEA_2 proteins are also closely associated to the resistance to multiple abiotic stresses.

## Conclusion

In this study, we identified 23 LEA proteins and their coding genes from Moso bamboo genome and classified them to six groups. We performed comparative analyses of structures, gene amount, biochemical characteristics, and evolutionary features of PeLEAs with those of *O*. *sativa* (OsLEAs), *B*. *distachyon* (BdLEAs), *Z*. *mays* (ZmLEAs), *S*. *bicolor* (SbLEAs), *Arabidopsis* (AtLEAs), and *P*. *trichocarpa* (PtLEAs). RNA-seq based expression profile revealed genes involved in responses to dehydration and cold stresses. The results present here provide comprehensive insights into the LEA family in *P*. *edulis* and the expression altas under dehydration and cold stresses, which will help to cope with the increasing environmental challenges in the future.

## Supporting Information

S1 Fig Correlation of expression levels of control and samples at each of time points between the two replicates. (TIF)

S1 Table List and biochemical characteristics of LEA in *P*. *edulis*, *O*. *sativa*, *B*. *distachyon*, *Z*. *mays*, and S. *bicolor*. (XLSX)

S2 Table Classifications of LEA proteins in different literatures. (XLSX)

S3 Table Homology to the known LEA proteins associated with stress tolerance. (XLSX)

S4 Table Paralogues of *PeLEAs* and orthologues in *O*. *sativa* and *B*. *distachyon*. (XLSX)

S5 Table The non-synonymous (Ka), synonymous (Ks) substitution rates and their ratios (Ka/Ks) of paralogous and orthologous gene pairs of *P*. *edulis*, *O*. *sativa*, and *B*. *distachyon*. (XLSX)
