# Supplementary figures and images for: Genome-Wide Identification, Characterization, and Stress-Responsive Expression Profiling of Genes Encoding LEA (Late Embryogenesis Abundant) Proteins in Moso Bamboo (Phyllostachys edulis)
Source: PLoS One. 2016 Nov 9;11(11):e0165953. doi: 10.1371/journal.pone.0165953 (PMC5102402; doi:10.1371/journal.pone.0165953)

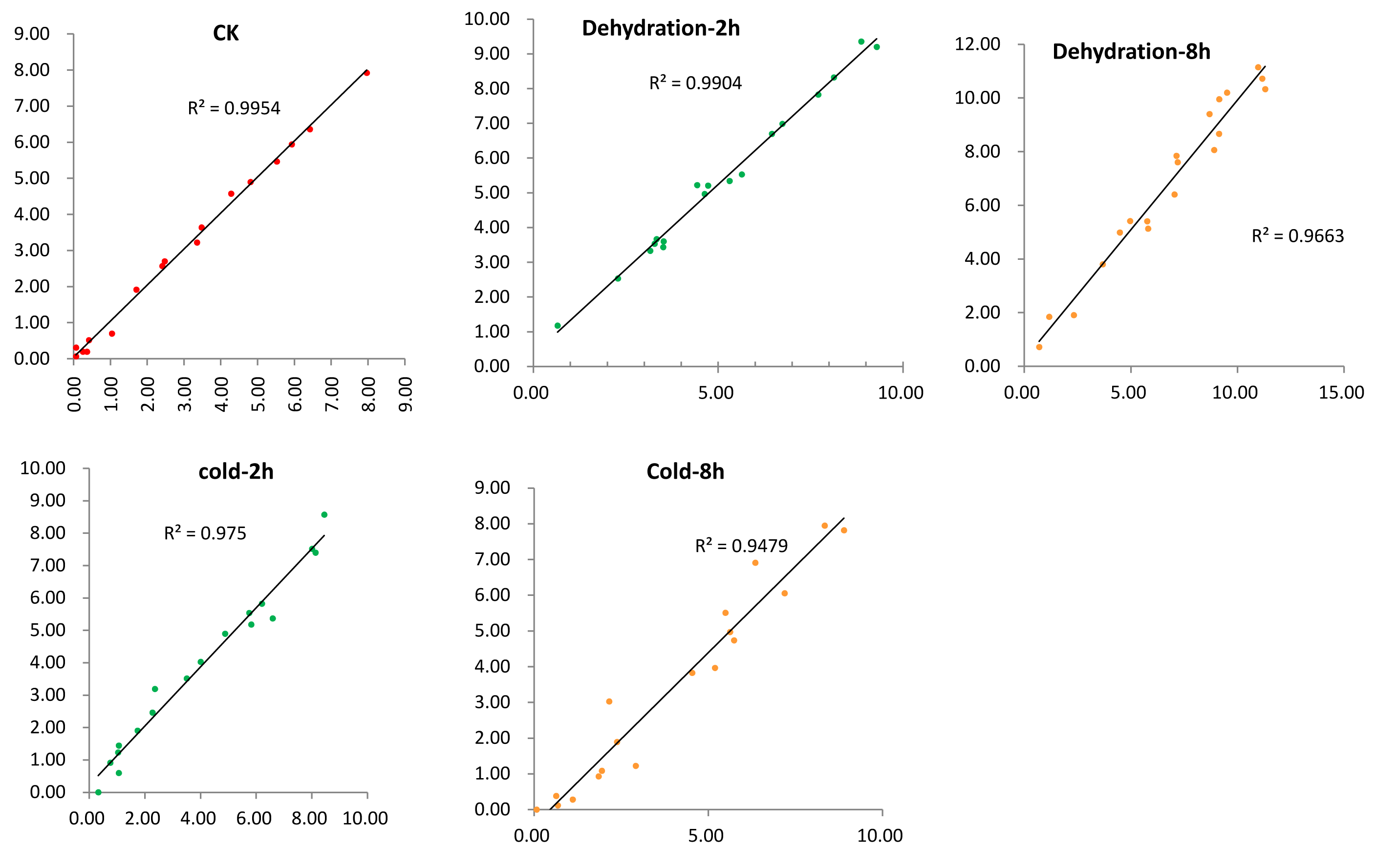

Supplement: S1 Fig — (TIF) [file pone.0165953.s001.tif]
